# Adverse Cardiovascular Outcomes in Patients With Syphilis

**DOI:** 10.1001/jamanetworkopen.2026.6771

**Published:** 2026-04-13

**Authors:** Eli Tsakiris, Han Feng, Ghassan Bidaoui, Christian Massad, Janice Zha, Zhihao Wu, Yishi Jia, Yingshuo Liu, Hadi Younes, Yara Menassa, Michel Abou Khalil, Mayana Bsoul, Mohammad Montaser Atasi, Samer Zakhour, David Mushatt, Omar Kreidieh, Nassir F. Marrouche, Amitabh C. Pandey

**Affiliations:** 1 Tulane Research Innovation for Arrhythmia Discovery, New Orleans, Louisiana; 2 Section of Cardiology, Heart and Vascular Institute, Department of Medicine, Tulane University School of Medicine, New Orleans, Louisiana; 3 Section of Infectious Diseases, Department of Medicine, Tulane University School of Medicine, New Orleans, Louisiana; 4 Department of Medicine, Southeast Louisiana Veterans Health Care System, New Orleans

## Abstract

**Importance:**

Syphilis, which is caused by *Treponema pallidum*, has historically been associated with cardiovascular complications. Despite a resurgence of syphilis incidence in the US, large-scale studies evaluating its independent cardiovascular impact are lacking.

**Objective:**

To determine whether syphilis infection is independently associated with increased risk of adverse cardiovascular outcomes in a US health system population.

**Design, Setting, and Participants:**

This retrospective cohort study with a 15-year follow-up (January 1, 2011, to July 1, 2025) was performed within a tertiary health care system in New Orleans, Louisiana. Participants included adults with syphilis identified using codes from the *International and Statistical Classification of Diseases, Tenth Revision* (*ICD-10*) and controls without syphilis who were matched based on demographic characteristics and comorbidities. Patients with preexisting cardiovascular disease were excluded. Data were analyzed from January 1, 2011, to July 1, 2025.

**Exposures:**

Diagnosis of syphilis (primary, secondary, tertiary, or unspecified stage) documented by *ICD-10* codes.

**Main Outcomes and Measures:**

Incident cardiovascular events included myocardial infarction, heart failure, aortic regurgitation, atrial fibrillation, aortic aneurysm or dissection, ischemic stroke, hemorrhagic stroke, peripheral artery disease, venous thromboembolism, and death. Outcomes were assessed using Kaplan-Meier survival curves and were adjusted for body mass index.

**Results:**

A total of 8814 participants were included in the analysis (4753 [53.9%] female; mean [SD] age, 50.0 [17.0] years). The syphilis group (n = 1469) and control group (n = 7345) were similar in terms of their demographic characteristics (mean [SD] age, 50.1 [17.0] vs 50.0 [17.6] years [*P* = .78]; 795 [54.1%] vs 3958 [53.9%] female [*P* = .84]) and comorbidities. In adjusted analyses, syphilis was associated with an increased risk of aortic aneurysm or dissection (hazard ratio [HR], 2.08; 95% CI, 1.47-2.94; *P* = .001), ischemic stroke (HR, 1.53; 95% CI, 1.27-1.84; *P* < .001), hemorrhagic stroke (HR, 1.92; 95% CI, 1.23-2.99; *P* = .004), peripheral artery disease (HR, 1.28; 95% CI, 1.01-1.62; *P* = .04), death (HR, 5.80; 95% CI, 3.81-8.82; *P* < .001), and myocardial infarction (HR, 1.33; 95% CI, 1.06-1.67; *P* = .01). No associations were found for heart failure (HR, 0.98; 95% CI, 0.83-1.17), atrial fibrillation (HR, 0.85; 95% CI, 0.67-1.08), aortic regurgitation (HR, 1.58; 95% CI, 0.87-2.87), or venous thromboembolism (HR, 1.47; 95% CI, 0.76-2.94).

**Conclusions and Relevance:**

In this retrospective cohort study of adults with syphilis, infection was independently associated with higher risk of several major cardiovascular outcomes. These findings underscore the importance of early detection and treatment of syphilis and suggest a role for incorporating cardiovascular risk assessment into comprehensive syphilis management strategies.

## Key Points

**Question:**

Is syphilis infection associated with an increased risk of adverse cardiovascular outcomes in a US health system population?

**Findings:**

In this cohort study of 1469 patients with syphilis matched with 7345 controls, syphilis was independently associated with higher risks of aortic aneurysm or dissection, ischemic and hemorrhagic stroke, peripheral artery disease, and myocardial infarction. Hazard ratios were greatest in patients with late-stage syphilis.

**Meaning:**

These findings suggest that syphilis infection may confer independent cardiovascular risk, supporting the need for early diagnosis, treatment, and consideration of cardiovascular risk assessment in syphilis management.

## Teaser

This cohort study assesses whether syphilis infection is independently associated with adverse cardiovascular outcomes in a US health system population without preexisting cardiovascular disease.

## Introduction

Cardiovascular disease is a leading cause of death globally, and traditional risk factors such as hypertension, diabetes, hyperlipidemia, smoking, and sedentary lifestyle have long been established as contributors to adverse outcomes. However, it should also be recognized that chronic infections and systemic inflammation may further contribute to an individual’s risk profile.^1,2^ Although early syphilis is often minimally symptomatic, untreated infection may progress to tertiary stages, which have historically been linked to cardiovascular sequelae. *Treponema pallidum* induces a chronic inflammatory response in the cardiovascular system, particularly targeting the vasa vasorum, which are smaller blood vessels that supply the metabolic needs of larger arteries, such as the aorta.^3^ This inflammation leads to vascular obliterations, characterized by endothelial cell swelling and perivascular lymphoplasmacytic infiltration, which compromise blood flow and result in ischemic damage.

Most data supporting cardiovascular effects of syphilis come from early autopsy series, international registries, or isolated case reports.^4,5,6^ To date, no US cohort studies, to our knowledge, have used matched controls to evaluate the independent association between syphilis and incident cardiovascular outcomes, resulting in a critical gap in this patient population. With syphilis resurging across the US and an increase of more than 50% in reported cases from 2018 to 2023, an investigation of the potential morbid effects of syphilis is important.^7,8^ The primary objective of this study was to assess the independent association of syphilis infection with adverse cardiovascular outcomes in a US health system population without preexisting cardiovascular disease.

## Methods

This cohort study was approved by the Tulane Institutional Review Board, which granted a waiver of informed consent because the study involved retrospective analyses of existing data and posed minimal risk to participants. We followed the Strengthening the Reporting of Observational Studies in Epidemiology (STROBE) reporting guideline.

### Study Population

This retrospective study used data extracted from structured electronic health records (EHRs) from 3 hospitals in New Orleans, Louisiana, within a single health care system with a 15-year follow-up between January 1, 2011, and July 1, 2025. All diagnoses and outcomes were determined through the record of relevant codes from *International and Statistical Classification of Diseases, Tenth Revision* (*ICD-10*). Patients 18 years or older were eligible for inclusion. Patients were excluded if key covariates required for matching and adjustment were missing (eg, age, sex, or body mass index) or if there was a documented prior occurrence of any primary cardiovascular outcome of interest before the index date to ensure that all outcomes represented incident events. The index date was defined as the first date of syphilis diagnosis for the study group or a corresponding baseline visit for matched controls. Stable cardiovascular comorbidities, such as nonobstructive coronary artery disease, were not exclusionary and were included as baseline covariates for matching and adjustment.

### Case Selection and Matching

Patients with syphilis were identified based on the documentation of the appropriate *ICD-10* code. A control cohort was selected from the unaffected population and matched based on age, sex, body mass index (BMI), and presence of diabetes, hypertension, hyperlipidemia, coronary artery disease, chronic obstructive pulmonary disease, chronic kidney disease, chronic liver disease, autoimmune disease, or cancer. Comorbidities were identified through a review of relevant *ICD-10* codes at the patient’s first encounter during the study period. Controls were matched 5:1 with patients with syphilis. Matching covariates were defined at or before the index date, with incident diagnoses occurring during follow-up not incorporated into the matching framework. To evaluate whether cardiovascular risk varied by disease progression, we analyzed subgroups of patients with syphilis by stage, as recorded in their EHR, in separate Cox proportional hazards regression analyses. Primary, secondary, tertiary, latent or unspecified syphilitic stages were identified using the relevant *ICD-10* codes, and patients with a record of multiple disease states were assigned to the subgroup corresponding to the most advanced documented disease stage. Each subgroup was compared with the original matched control cohort for each of the same primary outcomes. A comprehensive description of *ICD-10* codes used for syphilis staging, demographic characteristics, comorbidities, and outcomes is provided in eTables 1 to 4 in Supplement 1.

### Primary Outcomes

Primary outcomes comprised cardiovascular events after the index date, including acute myocardial infarction (MI), heart failure (HF), aortic regurgitation (AR), atrial fibrillation (AF), aortic aneurysm (AA) or aortic dissection (AD), ischemic stroke, hemorrhagic stroke, peripheral artery disease (PAD), and venous thromboembolism (VTE), identified using relevant *ICD-10* codes (eTable 2 in Supplement 1). Each cardiovascular outcome was modeled separately using outcome-specific time-to-event analyses. Participants were followed up from the index date until the first occurrence of the specific outcome, death, or end of follow-up on July 1, 2025. Each outcome was analyzed separately; participants were censored at the first event for that outcome.

### Statistical Analysis

Data were analyzed from January 1, 2011, to July 1, 2025. Continuous variables are presented as means (SDs) and were compared using 2-tailed *t* test or Wilcoxon test, depending on the normality check results based on Shapiro-Wilk tests. Categorical variables are expressed as frequencies and were compared using the χ^2^ test. Kaplan-Meier curves were generated to compare the cumulative incidence of patients with syphilis and unaffected controls, with the corresponding *P* values generated by log-rank tests. Univariable and multivariable Cox proportional hazards regression models were then applied to estimate the hazard ratio (HR) of syphilis for each cardiovascular outcome, with or without adjustment for other confounding variables. After propensity score matching, outcome-specific Cox proportional hazards regression models were fit with syphilis exposure as the primary covariate; because BMI remained imbalanced after matching, BMI was additionally included in all Cox proportional hazards regression models as a continuous covariate. All tests were 2 tailed, and *P* < .05 was considered statistically significant. The proportional hazards assumption for each Cox model was formally evaluated using Schoenfeld residual tests. Statistical analyses were performed using R, version 4.3.1 (R Project for Statistical Computing).

To assess the potential modifying effect of HIV infection, a subanalysis was performed in patients with HIV and syphilis, matched 1:1 with patients with HIV and no history of syphilis infection. Multivariable Cox proportional hazards regression models identical to those of the primary analysis were applied to this subgroup to evaluate HRs for each cardiovascular outcome. Cox proportional hazards regression models identical to the primary analysis with respect to measured outcomes and methodology were applied within this subgroup post hoc analysis.

## Results

### Demographics

**Figure 1.**
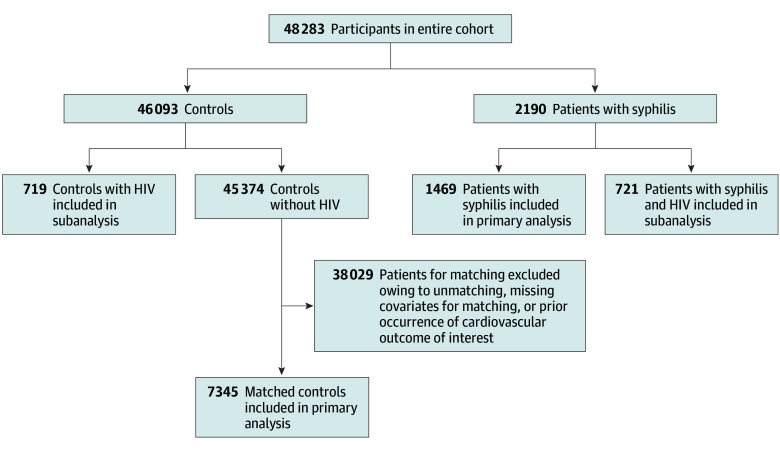
Flow Diagram of Cohort Selections

From a total population of 48 283 individuals, the matched cohorts included 8814 participants, of whom 7345 were controls and 1469 were patients with syphilis (4753 [53.9%] female and 4061 [46.1%] male; mean [SD] age, 50.0 [17.0] years). Among the 2190 participants with syphilis, all extracted variables, including age, sex, BMI, and diagnosis information were complete, with no missing data. A flow diagram of cohort selection, including the HIV subanalysis selection, is shown in Figure 1. In the control group and syphilis group, the mean (SD) age (50.0 [17.6] vs 50.1 [17.0] years, respectively; *P* = .78) and sex distribution (female, 3958 [53.9%] vs 795 [54.1%], respectively; male, 3387 [46.1%] vs 674 [45.9%], respectively; *P* = .84) were similar. BMI (calculated as weight in kilograms divided by height in meters squared) was lower in the syphilis group than in the control group (mean [SD], 24.9 [7.0] vs 28.1 [8.6]; *P* < .001). The mean (SD) follow-up time for the overall cohort was 2337 (1398) days, with a median follow-up time of 2388 (IQR, 1158-3396) days after matching. Baseline rates of other preselected comorbidities were similar between groups, as shown in Table 1, with incident comorbid diagnoses during follow-up summarized in eTable 5 in Supplement 1.

**Table 1.** Baseline Demographic and Clinical Characteristics of the Study Groups^a^

Characteristic	Study group, No. (%)		*P* value
	Controls (n = 7345)	Patients with syphilis (n = 1469)	
Age, mean (SD), y	50.0 (17.6)	50.1 (17.0)	.78
Sex			
Female	3958 (53.9)	795 (54.1)	.84
Male	3387 (46.1)	674 (45.9)	
BMI, mean (SD)	28.1 (8.6)	24.9 (7.0)	<.001
Diabetes	2072 (28.2)	404 (27.5)	.60
Hypertension	1256 (17.1)	263 (17.9)	.47
Hyperlipidemia	2736 (37.2)	538 (36.6)	.64
Coronary artery disease	712 (9.7)	147 (10.0)	.65
Chronic obstructive pulmonary disease	690 (9.4)	147 (10.0)	.51
Chronic kidney disease	1050 (14.3)	228 (15.5)	.29
Chronic liver disease	896 (12.2)	182 (12.4)	.82
Autoimmune disease	470 (6.4)	97 (6.6)	.74
Cancer	647 (8.8)	137 (9.3)	.51

Abbreviation: BMI, body mass index (calculated as weight in kilograms divided by height in meters squared).

^a^ Matching was performed to control for key cardiovascular risk factors. No statistically significant differences were observed for age, sex, or major comorbidities aside from BMI, which was slightly lower in the syphilis group.

### Cardiovascular Outcomes: Event Rates

**Figure 2.**
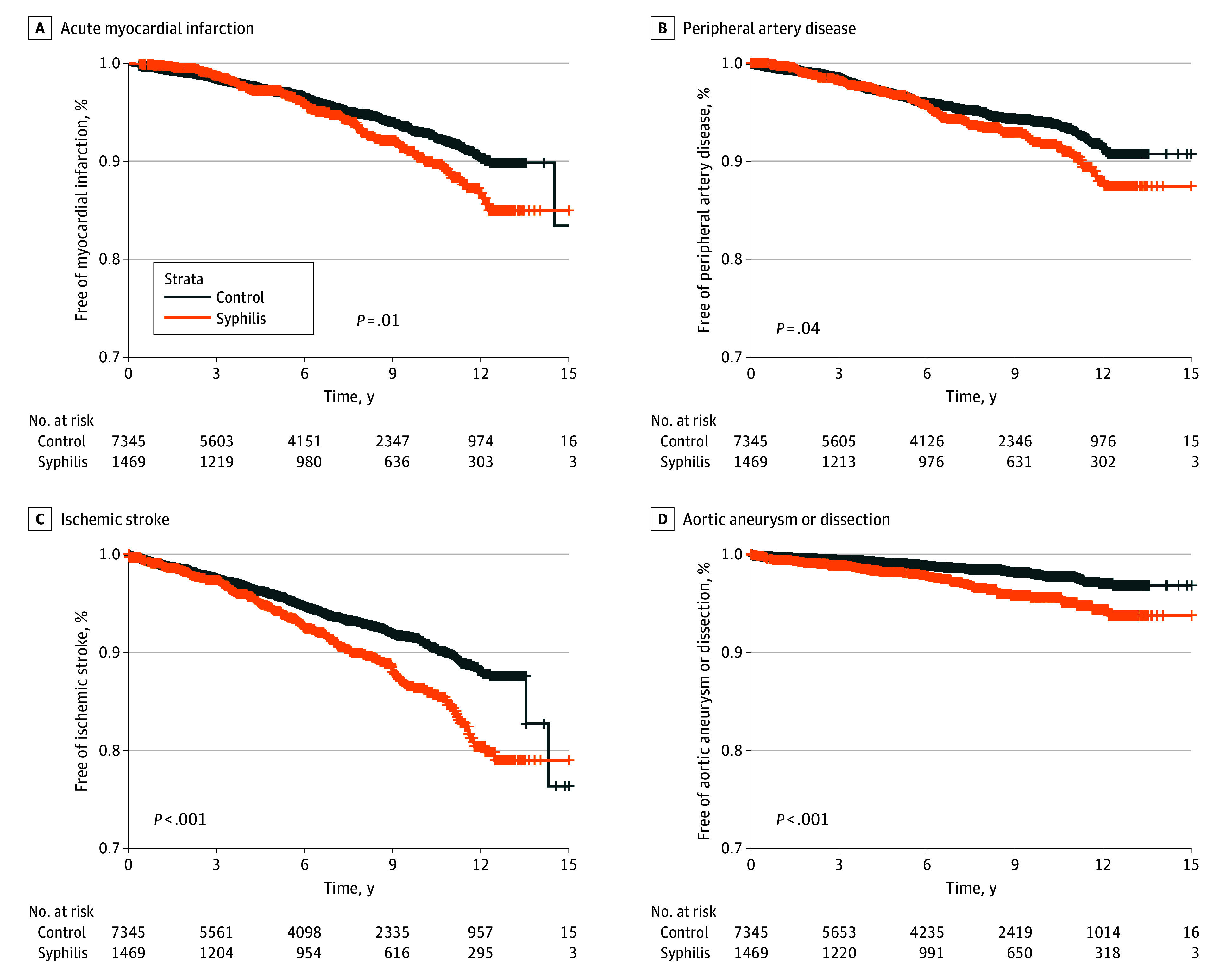
Kaplan-Meier Survival Curves for Cardiovascular Outcomes

Patients with syphilis experienced significantly higher rates of several cardiovascular events compared with matched controls within the study period. MI occurred in 101 patients with syphilis (6.9%) vs 311 controls (4.2%) (*P* < .001), while ischemic stroke occurred in 152 patients with syphilis (10.3%) vs 421 controls (5.7%) (*P* < .001). Hemorrhagic stroke was also more common in the syphilis group (29 [2.0%] vs 60 [0.8%]; *P* < .001). Vascular complications such as AA or AD occurred in 48 patients with syphilis (3.3%) compared with 97 controls (1.3%) (*P* < .001), and PAD developed in 91 patients with syphilis (6.2%) vs 299 controls (4.1%) (*P* < .001). AR was also more frequent in the syphilis group (15 [1.0%] vs 40 [0.5%]; *P* = .03). There were no statistically significant differences in the rates of HF (164 [11.2%] vs 706 [9.6%]; *P* = .07), VTE (12 [0.8%] vs 33 [0.4%]; *P* = .07), or AF (79 [5.4%] vs 393 [5.4%]; *P* = .97). Comprehensive event rates are provided in Table 2. Kaplan-Meier curves for MI, PAD, ischemic stroke, and AA or AD are shown in Figure 2. Kaplan-Meier curves for all other primary outcomes are included in eFigures 1 to 6 in Supplement 1.

**Table 2.** Cardiovascular Outcomes by Study Group

Outcome	Study group, No. (%)		*P* value
	Controls (n = 7345)	Patients with syphilis (n = 1469)	
Acute myocardial infarction	311 (4.2)	101 (6.9)	<.001
Heart failure	706 (9.6)	164 (11.2)	.07
Aortic regurgitation	40 (0.5)	15 (1.0)	.03
Atrial fibrillation	393 (5.4)	79 (5.4)	.97
Aneurysm or dissection	97 (1.3)	48 (3.3)	<.001
Ischemic stroke	421 (5.7)	152 (10.3)	<.001
Hemorrhagic stroke	60 (0.8)	29 (2.0)	<.001
Venous thromboembolism	33 (0.4)	12 (0.8)	.07
Peripheral artery disease	299 (4.1)	91 (6.2)	<.001

### Cardiovascular Outcomes: Multivariable Analysis

**Figure 3.**
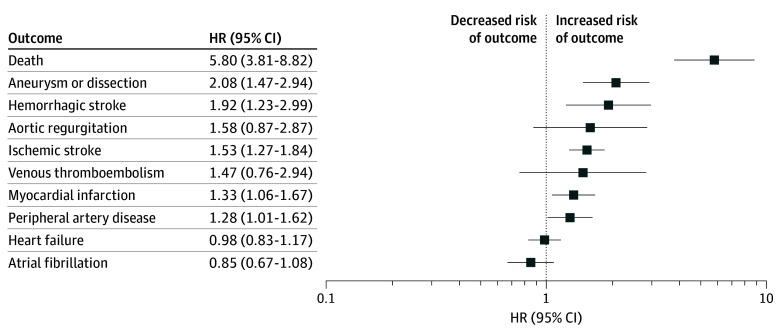
Forest Plot of Hazard Ratios (HRs) for Cardiovascular Outcomes

To account for possible confounders, Cox proportional hazards regression analysis was performed to investigate the independent association of syphilis infection with cardiovascular outcomes between the matched cohorts. Patients with syphilis had a significantly higher risk of AA or AD (HR, 2.08; 95% CI, 1.47-2.94; *P* = .001), ischemic stroke (HR, 1.53; 95% CI, 1.27-1.84; *P* < .001), hemorrhagic stroke (HR, 1.92; 95% CI, 1.23-2.99; *P* = .004), PAD (HR, 1.28; 95% CI, 1.01-1.62; *P* = .04), MI (HR, 1.33; 95% CI, 1.06-1.67; *P* = .01), and death (HR, 5.80; 95% CI, 3.81-8.82; *P* < .001) compared with controls. HF (HR, 0.98; 95% CI, 0.83-1.17), AF (HR, 0.85; 95% CI, 0.67-1.08), AR (HR, 1.58; 95% CI, 0.87-2.87), and VTE (HR, 1.47; 95% CI, 0.76-2.94) did not differ significantly between groups on multivariable analysis (Figure 3). Proportional hazards violations were observed for HF, AF, and hemorrhagic stroke (eTable 6 in Supplement 1). Full multivariable results are found in the eTable 7 in Supplement 1.

### Subanalysis: Outcomes by Syphilis Stage

In primary syphilis (n = 59) and secondary syphilis (n = 67), no associations were observed for any primary cardiovascular outcomes. Some outcomes (eg, hemorrhagic stroke and AR) were not estimable due to sparse events. In contrast, tertiary syphilis (n = 195) demonstrated increased rates of mortality (HR, 6.93; 95% CI, 3.98-12.08; *P* < .001), AA or AD (HR, 5.57; 95% CI, 3.40–9.13; *P* < .001), ischemic stroke (HR, 3.23; 95% CI, 2.36-4.43; *P* < .001), hemorrhagic stroke (HR, 2.62; 95% CI, 1.14-6.00; *P* = .02), MI (HR, 2.15; 95% CI, 1.40-3.30; *P* = .001), PAD (HR, 2.45; 95% CI, 1.61-3.73; *P* < .001), HF (HR, 2.01; 95% CI, 1.47-2.74; *P* < .001), and AF (HR, 2.19; 95% CI, 1.46-3.28; *P* < .001). Rates of AR (HR, 2.20; 95% CI, 0.69-7.03; *P* = .19) and VTE (HR, 0.84; 95% CI, 0.12-6.09; *P* = .86) did not demonstrate statistically significant increases.

In patients with latent syphilis, diagnoses of early latent syphilis occurred less frequently (n = 20) and showed no associations across evaluated outcomes, with several outcomes not estimable due to sparse events. Patients with late latent syphilis (n = 327) had significantly increased mortality (HR, 4.08; 95% CI, 2.37-7.00; *P* < .001), AA or AD (HR, 2.41; 95% CI, 1.41-4.11; *P* = .001), and ischemic stroke (HR, 1.82; 95% CI, 1.34-2.47; *P* < .001). Associations were not present for hemorrhagic stroke, MI, PAD, HF, AF, VTE or AR. Comprehensive multivariable Cox proportional hazards regression models for each syphilis subgroup can be found in eTables 8 to 13 in Supplement 1.

### Subanalysis: HIV Coinfection

In the post hoc subgroup analysis of patients with syphilis and comorbid HIV infection (n = 721) compared with a control cohort with HIV (n = 719), syphilis was not associated with a significantly increased risk of most cardiovascular outcomes after matching. HRs for AA or AD (0.75; 95% CI, 0.37-1.52), hemorrhagic stroke (0.95; 95% CI, 0.40-2.23), ischemic stroke (1.06; 95% CI, 0.68-1.66), MI (0.99; 95% CI, 0.64-1.54), PAD (0.97; 95% CI, 0.55-1.73), HF (0.93; 95% CI, 0.65-1.32), AF (0.81; 95% CI, 0.45-1.47), VTE (1.24; 95% CI, 0.38-4.06), and AR (1.51; 95% CI, 0.25-9.03) did not demonstrate a statistically significant increase.

In contrast, all-cause mortality remained significantly elevated among patients with both syphilis and HIV (HR, 11.32; 95% CI, 3.47-36.91). A forest plot of estimated HR for this subanalysis is provided in eFigure 7 in Supplement 1.

## Discussion

In this retrospective cohort study, syphilis was independently associated with higher risk of AA or AD, ischemic and hemorrhagic stroke, PAD, MI, and death, with the highest risk in late-stage disease. Syphilis incidence in the US climbed by more than 80% from 2018 to 2022, reaching more than 200 000 reported cases in 2023.^8^ Southern states have even higher incidence (74 vs 64 cases per 100 000 nationally). Louisiana ranks among the highest at 101 cases per 100 000, similar to the incidence of 113 per 100 000 in our cohort from New Orleans.^9^ The Louisiana Department of Health reported that late or unknown stages of syphilis rose by 28% in New Orleans between 2018 and 2023, with 80.6 cases per 100 000 population of late syphilis or syphilis of unknown stage in 2023.^10^ Given this sustained resurgence, our findings suggest that routine cardiovascular risk assessment (eg, blood pressure, lipid levels, and ankle-brachial index screening) should be considered as part of comprehensive syphilis management. In particular, patients with a history of syphilis may benefit from discussion about aggressive treatment of cardiovascular risks, given the increased risk associated with cardiovascular adverse outcomes with late or untreated infection. Conversely, clinicians caring for patients with established cardiovascular disease may also consider syphilis testing in appropriate clinical contexts, as this could facilitate identification and treatment of undiagnosed infection. Additional data are needed before formal recommendations can be made regarding cardiovascular screening in patients with syphilis or syphilis testing in patients with cardiovascular disease. The role of treating the underlying infection in preventing cardiovascular disease remains unclear. Additionally, clinicians evaluating patients with established or unexplained vascular pathology (particularly aortic disease) may consider syphilis testing in appropriate clinical contexts following a thorough social history, although prospective data are needed before routine screening can be recommended.

### Population Studies of Syphilis as a Cardiovascular Risk Factor

Modern large population studies examining the association of cardiovascular outcomes with syphilis in the US are minimal. In the Tuskegee Syphilis Study, 16% of autopsied participants had thoracic aneurysms or coronary ostial narrowing.^11,12^ Pre–penicillin era autopsies in Philadelphia from 1927 to 1937 yielded a cardiovascular sequelae rate of 6.9%,^13^ whereas the results of a post–penicillin era series from New York from 1950 to 1960 reported that syphilitic aortitis rates fell to 0.8%, highlighting the population-level benefit of treatment.^14^ More recently, syphilis has been studied predominantly through case studies demonstrating unique presentations, necessitating further larger studies to underscore the importance of screening and treatment. Additionally, new efforts focusing on primary cardiovascular prevention and more aggressive recommendations for blood pressure control may have mitigated some of the cardiovascular responses previously reported.^15,16,17^

Due to the scarcity of evidence, our understanding of population-level association of syphilis with cardiovascular outcomes in the US is informed mainly by international studies. A study of untreated syphilis in 2000 patients in Oslo, Norway, in the 1950s^18^ found that 13.6% of males and 7.6% of females eventually developed cardiovascular manifestations of the infection, with aortic pathology in 4.6% of patients. More modern retrospective cohort studies in Italy^19^ and Canada,^20^ looking broadly at tertiary syphilis, also reported lower rates of cardiovascular events, again supporting the introduction of penicillin as a turning point for syphilis treatment. Most recently, Wu et al^21^ presented a national report of 28 796 patients with syphilis between 2010 and 2015 in Taiwan that demonstrated increased rates of cardiovascular comorbidity and a relative risk of 155% for cardiovascular death in patients with syphilis compared with matched controls. These results are similar to our findings for MI, ischemic stroke, and aortic pathology. Wu et al^21^ also found a small but statistically significant risk of all cardiovascular outcomes analyzed, including VTE, AF, and HF, which was not observed in our analysis. Notably, the Taiwan-based study did not evaluate PAD as an outcome or attempt to stratify patients by syphilis disease stage, and it had a short follow-up of 5 years.

We also found a significantly higher risk for mortality, although this finding warrants careful consideration. Mortality risk was most pronounced in patients with late-stage disease, consistent with the known natural history of syphilis and its potential for progressive vascular and multiorgan damage. Although it has been reported that as many as 10% of patients who are not treated for syphilis may die from the infection, residual confounding likely also contributes to the magnitude of the mortality association found in our analysis.^22^ While the mortality signal is plausible and influenced largely by outcomes in patients with tertiary syphilis, it should not be interpreted as direct causal mortality from syphilis alone.

In the post hoc subgroup analysis restricted to patients with HIV, the risks for most cardiovascular outcomes were attenuated and no longer statistically significant, with the exception of all-cause mortality. Likely, in patients with HIV, in whom baseline cardiovascular risk is already elevated, it is difficult to parse any additive effect that syphilis may have.^23^ Larger studies are needed to clarify whether HIV status meaningfully modifies the cardiovascular risk associated with syphilis. Our findings after a 15-year follow-up contribute to growing data on the cardiovascular hazard of syphilis analysis by examining a US population with increased comorbidity and infection rates in Louisiana. Furthermore, these data can be extended to the southern region of the US, as well as to some degree to the entire US.

### Association of Late Syphilis With Cardiovascular Outcomes

To our knowledge, this study is the first modern US retrospective cohort analysis to identify an independent association between syphilis and cardiovascular outcomes since the Tuskegee Syphilis Study, in addition to being the first study to examine the association of syphilis with the development of PAD using matched controls. PAD is increasingly recognized as prognostically equivalent to coronary artery disease, as patients with PAD have a markedly elevated risk of MI, stroke, and mortality.^24,25,26^ It is accepted that *T pallidum* may cause systemic vascular inflammation, which in turn results in the association with many of the aortopathies previously described.^27,28^ However, we found an association with not only MI (which is suggestive of possible involvement of the coronary arteries), but also PAD. It would follow that, given the systemic nature of inflammation that occurs with syphilis, the vasculature of all calibers would be subjected to the negative influence. While there are case studies of PAD in patients with syphilis, most previous literature has focused on aortic aneurysms or coronary involvement, with PAD being overlooked in the larger retrospective studies.^29,30,31^

Since the mechanism of syphilis’s effect on cardiovascular outcomes is believed to be through vascular obliteration, it is reasonable that our study found a more profound association with conditions that are more immediately related to vasculature (eg, aortic pathology, MI, arterial disease, ischemic and hemorrhagic stroke) as opposed to diseases of muscular tissue (eg, HF or AF), valvular tissue (eg, AR), or thrombus formation (eg, VTE). Further, our subanalysis findings strengthen evidence that syphilis causes adverse cardiovascular outcomes in later infection stages. It remains unclear the extent to which early and latent infection influence cardiovascular outcomes, as the progression of syphilis from primary to secondary and later stages is unpredictable and does not occur on a consistent timeline for all patients. This heterogeneity in disease trajectory complicates the interpretation of cardiovascular risks and underscores the need for early identification and treatment before vascular damage occurs.

### Limitations

Our study has inherent limitations due to its retrospective design. Although an attempt was made to mitigate differences between study groups through comprehensive matching, causation cannot be inferred. Additionally, although care was taken to include relevant comorbidity and demographic variables in propensity matching, some variables, including smoking status and race and ethnicity, are not accounted for and may be unequally distributed between study groups. Other variables (eg, HF) may have heterogeneity between disease states. Events outside the health system could not be accounted for. A large proportion of patients were classified with *ICD-10* codes for unspecified syphilis, a group that showed inconsistent or inverse associations with cardiovascular outcomes. These codes likely reflect documentation gaps, and while the lack of clear association with cardiovascular outcomes makes it more likely that they included patients with early-stage (active or latent) disease, it cannot be definitively stated that this cohort included patients with advanced disease. Importantly, misclassification may also apply to the comparison group, as individuals without documented syphilis diagnoses may include patients with undiagnosed or previously treated infection not captured in structured EHR data. Laboratory data (eg, rapid plasma reagin titers) and treatment (eg, with benzathine penicillin G) were unable to be obtained, limiting the ability to discern active infection from treated infection or to evaluate the importance of treatment in preventing cardiovascular outcomes. Additional longitudinal studies with longer follow-up times will continue to help improve understanding and further delineate the temporality of cardiovascular risks of syphilis infection.

## Conclusions

This retrospective cohort study found that syphilis was independently associated with increased risk of several adverse cardiovascular outcomes, particularly those involving vascular tissue, such as AA or AD, ischemic and hemorrhagic stroke, PAD, and MI. Given the rising incidence of syphilis both locally and nationally, these findings underscore the importance of early detection and treatment. Further research is needed to understand whether prompt antibiotic therapy mitigates long-term cardiovascular risk and to identify the best screening practices in patients most vulnerable to cardiovascular complications.

## Notes

Supplement 1. **eTable 1.** *ICD-10* Codes for Demographic Characteristics and Comorbidities **eTable 2.** *ICD-10* Codes for Cardiovascular Outcomes **eTable 3.** *ICD-10* Codes for Exclusion Criteria (History of CVD or HIV) **eTable 4.** *ICD-10* Codes for Syphilis Staging (Primary, Secondary, Tertiary, or Unspecified) **eTable 5.** Incident Comorbid Diagnoses During Follow-up in the Matched Cohorts **eTable 6.** Schoenfeld Residual Tests Assessing Proportional Hazards Assumptions for Primary Cox Regression Models **eTable 7.** Cox Regression Analyses for All Syphilis Cases (Cardiovascular Outcomes) **eTable 8.** Cox Regression Analyses for Tertiary Syphilis **eTable 9.** Cox Regression Analyses for Secondary Syphilis **eTable 10.** Cox Regression Analyses for Primary Syphilis **eTable 11.** Cox Regression Analyses for Syphilis, Unspecified **eTable 12.** Cox Regression Analyses for Syphilis, Early Latent **eTable 13.** Cox Regression Analyses for Syphilis, Late Latent **eFigure 1.** Kaplan Meier Survival Curve for Heart Failure, Primary Analysis **eFigure 2.** Kaplan Meier Survival Curve for Atrial Fibrillation, Primary Analysis **eFigure 3.** Kaplan Meier Survival Curve for Aortic Regurgitation, Primary Analysis **eFigure 4.** Kaplan Meier Survival Curve for Hemorrhagic Stroke, Primary Analysis **eFigure 5.** Kaplan Meier Survival Curve for Venous Thromboembolism, Primary Analysis **eFigure 6.** Kaplan Meier Survival Curve for Mortality, Primary Analysis **eFigure 7.** Forest Plot of Estimated Hazard Ratios, HIV Subanalysis

Supplement 2.
Data Sharing Statement
