# Supplementary material for: Adverse Cardiovascular Outcomes in Patients With Syphilis
Source: JAMA Netw Open. 2026 Apr 13;9(4):e266771. doi: 10.1001/jamanetworkopen.2026.6771 (PMC13077513; doi:10.1001/jamanetworkopen.2026.6771)
Supplement: Supplement 1. — eTable 1. ICD-10 Codes for Demographic Characteristics and Comorbidities eTable 2. ICD-10 Codes for Cardiovascular Outcomes eTable 3. ICD-10 Codes for Exclusion Criteria (History of CVD or HIV) eTable 4. ICD-10 Codes for Syphilis Staging (Primary, Secondary, Tertiary, or Unspecified) eTable 5. Incident Comorbid Diagnoses During Follow-up in the Matched Cohorts eTable 6. Schoenfeld Residual Tests Assessing Proportional Hazards Assumptions for Primary Cox Regression Models eTable 7. Cox Regression Analyses for All Syphilis Cases (Cardiovascular Outcomes) eTable 8. Cox Regression Analyses for Tertiary Syphilis eTable 9. Cox Regression Analyses for Secondary Syphilis eTable 10. Cox Regression Analyses for Primary Syphilis eTable 11. Cox Regression Analyses for Syphilis, Unspecified eTable 12. Cox Regression Analyses for Syphilis, Early Latent eTable 13. Cox Regression Analyses for Syphilis, Late Latent eFigure 1. Kaplan Meier Survival Curve for Heart Failure, Primary Analysis eFigure 2. Kaplan Meier Survival Curve for Atrial Fibrillation, Primary Analysis eFigure 3. Kaplan Meier Survival Curve for Aortic Regurgitation, Primary Analysis eFigure 4. Kaplan Meier Survival Curve for Hemorrhagic Stroke, Primary Analysis eFigure 5. Kaplan Meier Survival Curve for Venous Thromboembolism, Primary Analysis eFigure 6. Kaplan Meier Survival Curve for Mortality, Primary Analysis eFigure 7. Forest Plot of Estimated Hazard Ratios, HIV Subanalysis [file jamanetwopen-e266771-s001.pdf]

Supplementary Online Content

Tsakiris E, Feng H, Bidaoui G, et al. Adverse cardiovascular outcomes in patients with syphilis. *JAMA Netw Open.* 2026;9(4):e266771. doi:10.1001/jamanetworkopen.2026.6771

eTable 1. *ICD-10* Codes for Demographic Characteristics and Comorbidities
eTable 2. *ICD-10* Codes for Cardiovascular Outcomes
eTable 3. *ICD-10* Codes for Exclusion Criteria (History of CVD or HIV)
eTable 4. *ICD-10* Codes for Syphilis Staging (Primary, Secondary, Tertiary, or Unspecified)
eTable 5. Incident Comorbid Diagnoses During Follow-Up in the Matched Cohorts
eTable 6. Schoenfeld Residual Tests Assessing Proportional Hazards Assumptions for Primary Cox Regression Models
eTable 7. Cox Regression Analyses for All Syphilis Cases (Cardiovascular Outcomes)
eTable 8. Cox Regression Analyses for Tertiary Syphilis
eTable 9. Cox Regression Analyses for Secondary Syphilis
eTable 10. Cox Regression Analyses for Primary Syphilis
eTable 11. Cox Regression Analyses for Syphilis, Unspecified
eTable 12. Cox Regression Analyses for Syphilis, Early Latent
eTable 13. Cox Regression Analyses for Syphilis, Late Latent
eFigure 1. Kaplan Meier Survival Curve for Heart Failure, Primary Analysis
eFigure 2. Kaplan Meier Survival Curve for Atrial Fibrillation, Primary Analysis
eFigure 3. Kaplan Meier Survival Curve for Aortic Regurgitation, Primary Analysis
eFigure 4. Kaplan Meier Survival Curve for Hemorrhagic Stroke, Primary Analysis
eFigure 5. Kaplan Meier Survival Curve for Venous Thromboembolism, Primary Analysis
eFigure 6. Kaplan Meier Survival Curve for Mortality, Primary Analysis
eFigure 7. Forest Plot of Estimated Hazard Ratios, HIV Subanalysis

This supplementary material has been provided by the authors to give readers additional information about their work.

© 2026 Tsakiris E et al. *JAMA Network Open*.

eTable 1. *ICD-10* Codes for Demographic Characteristics and Comorbidities

Condition	ICD-10 Codes
Diabetes Mellitus	E08.x, E09.x, E10.x, E11.x, E13.x
Hypertension	I10.x, I11.x, I12.x, I13.x
Hyperlipidemia	E78.x
Coronary Artery Disease	I25.10, I25.11, I20.x
Chronic Obstructive Pulmonary Disease (COPD)	J41.x, J42, J43.x, J44.x
Chronic Kidney Disease (CKD)	N18.1–N18.6, N18.9, Z99.2
Chronic Liver Disease	K70.3x, K74.6x, K76.0, B18.x
Autoimmune Diseases	M05.x, M32.x, M35.0x, K50.x, K51.x, E05.x, E06.3, L40.x
Cancer	C00–C97, C80.1, Z85.x

Table 1: ICD-10 codes used to define key cardiovascular and systemic comorbidities in the study population for matching and adjustment, including diabetes, hypertension, hyperlipidemia, coronary artery disease, and other relevant chronic conditions.

© 2026 Tsakiris E et al. *JAMA Network Open*.

eTable 2. *ICD-10* Codes for Cardiovascular Outcomes

Outcome	ICD-10-CM Codes
Acute myocardial infarction	I20.x, I21.x, I22.x, I24.x
Heart Failure	I50.x
Aortic regurgitation	I35.1
Aneurysm / Dissection of aorta	A52.01, A52.19, H35.041, H35.042, H35.043, H35.049, I25.41, I25.42, I28.1, I67.0, I67.1, I71.x, I72.x, I77.7, I77.89, I79.x
Atrial fibrillation	I48.0, I48.1, I48.2, I48.91
Ischemic Stroke	I63.x, I65.x, I66.x, I67.x, I68.x, G45.x, G46.x
Hemorrhagic Stroke	I60.x, I61.x, I62.x
Venous thromboembolism	I80.0-I80.3, I82.9, O22.2 – O22.3, O87.0 – O87.1, I26.0, I26.9
Peripheral artery disease	I77.6, I73.9

Table 2: ICD-10 codes used to identify primary cardiovascular outcomes of interest, including myocardial infarction, stroke subtypes, heart failure, aortic pathology, atrial fibrillation, venous thromboembolism, and non-atherosclerotic peripheral artery disease.

© 2026 Tsakiris E et al. *JAMA Network Open*.

eTable 3. *ICD-10* Codes for Exclusion Criteria (History of CVD or HIV)

Condition	ICD-10 Code(s)
History of Myocardial Infarction	I25.2
History of Heart Failure	Z86.79
History of Aortic Regurgitation	I35.1
History of Aneurysm or Dissection	Z86.79
Ischemic Stroke	I69.3x
Hemorrhagic Stroke	I69.0x / I69.1x
Venous Thromboembolism	Z86.718
Transient Ischemic Attack	Z86.73
HIV	B20, Z21, R75

Table 3: ICD-10 codes used to exclude patients with pre-existing cardiovascular disease or HIV infection at baseline, ensuring a cohort without prior events to better assess incident cardiovascular outcomes.

© 2026 Tsakiris E et al. *JAMA Network Open*.

eTable 4. *ICD-10* Codes for Syphilis Staging (Primary, Secondary, Tertiary, or Unspecified)

Syphilis Stage	ICD-10 Codes
**Primary Syphilis**	A51.0, A51.1, A51.2
**Secondary Syphilis**	A51.3, A51.4
**Early Latent Syphilis**	A51.5
**Late Latent Syphilis**	A52.8
**Tertiary Syphilis**	A52.0, A52.1, A52.3, A52.7
**Unspecified Syphilis**	A53.0, A53.9

Table 4: ICD-10 codes used to categorize syphilis cases by clinical stage, enabling stratified analysis of cardiovascular risk by disease progression.

© 2026 Tsakiris E et al. *JAMA Network Open*.

Table 5. Incident Comorbid Diagnoses During Follow-up in the Matched Cohorts

Incident diagnosis during follow-up*	No. (%)
Diabetes mellitus	309 (3.5)
Hypertension	97 (1.1)
Hyperlipidemia	218 (2.5)
Coronary artery disease	89 (1.0)
Chronic obstructive pulmonary disease	107 (1.2)
Chronic kidney disease	115 (1.3)
Chronic liver disease	131 (1.5)
Autoimmune disease	93 (1.1)
Cancer	109 (1.2)

Supplemental Table 5: Incident diagnoses after the index date among participants in the matched cohort (1,469 syphilis; 7,345 controls). Baseline comorbidities were defined at or before the index date and were used for propensity score matching; incident diagnoses during follow-up were not treated as time-varying covariates and were not incorporated into matching.

© 2026 Tsakiris E et al. *JAMA Network Open*.

eTable 6. Schoenfeld Residual Tests Assessing Proportional Hazards Assumptions for Primary Cox Regression Models

Outcome	p-value for Schoenfeld residual test
Acute Myocardial Infarction	0.05
Heart Failure	0.004
Aortic Regurgitation	0.17
Atrial Fibrillation	0.0011
Aneurysm or Dissection	0.92
Ischemic Stroke	0.051
Hemorrhagic Stroke	0.016
Venous Thromboembolism	0.13
Peripheral Artery Disease	0.12
Death	0.27

Table 6. Schoenfeld residual tests assessing proportional hazards assumptions for primary Cox regression models.

© 2026 Tsakiris E et al. *JAMA Network Open*.

eTable 7. Cox Regression Analyses for All Syphilis Cases (Cardiovascular Outcomes)

Outcome	HR	95% CI	P Value
Death	5.80	3.81-8.82	**<0.001**
Acute Myocardial Infarction	1.31	1.06-1.66	**0.01**
Heart Failure	0.98	0.82-1.17	0.84
Aortic Regurgitation	1.58	0.87-2.87	0.13
Atrial Fibrillation	0.85	0.67-1.08	0.19
Aneurysm or Dissection	2.08	1.47-2.91	**<0.001**
Ischemic Stroke	1.53	1.27-1.84	**<0.001**
Hemorrhagic Stroke	1.92	1.23-2.99	**0.004**
Venous Thromboembolism	1.47	0.76-2.82	0.26
Peripheral Artery Disease	1.28	1.01-1.62	**0.04**

Table 7: Hazard ratios, confidence intervals, and p-values from multivariable Cox regression models evaluating the association between syphilis and each cardiovascular outcome. Syphilis was independently associated with increased hazard for aneurysm/dissection, ischemic stroke, hemorrhagic stroke, death and peripheral artery disease after adjustment for baseline covariates.

© 2026 Tsakiris E et al. *JAMA Network Open*.

eTable 8. Cox Regression Analyses for Tertiary Syphilis

Outcome	Hazard Ratio	95% Confidence Interval	P Value
Death	6.93	3.98–12.08	<0.001
Aortic Aneurysm/Dissection	5.57	3.40–9.13	<0.001
Hemorrhagic Stroke	2.62	1.14–6.00	0.02
Aortic Regurgitation	2.20	0.69–7.03	0.19
Ischemic Stroke	3.23	2.36–4.43	<0.001
Venous Thromboembolism	0.84	0.12–6.09	0.86
Acute Myocardial Infarction	2.15	1.40–3.30	0.001
Peripheral Artery Disease	2.45	1.61–3.73	<0.001
Heart Failure	2.01	1.47–2.74	<0.001
Atrial Fibrillation	2.19	1.46–3.28	<0.001

Table 8: Multivariable Cox regression analyses evaluating associations between tertiary syphilis and cardiovascular outcomes

© 2026 Tsakiris E et al. *JAMA Network Open*.

eTable 9. Cox Regression Analyses for Secondary Syphilis

Outcome	Hazard Ratio	95% Confidence Interval	P Value
Death	1.15	0.16–8.28	0.889
Aortic Aneurysm/Dissection	0.79	0.11–5.63	0.812
Hemorrhagic Stroke	2.54	0.63–10.33	0.192
Aortic Regurgitation	NA	—	0.995
Ischemic Stroke	0.80	0.30–2.14	0.654
Venous Thromboembolism	NA	—	0.996
Acute Myocardial Infarction	0.54	0.13–2.15	0.377
Peripheral Artery Disease	0.29	0.04–2.05	0.214
Heart Failure	0.25	0.06–1.02	0.053
Atrial Fibrillation	NA	—	0.987

Table 9: Multivariable Cox regression analyses evaluating associations between secondary syphilis and cardiovascular outcomes

© 2026 Tsakiris E et al. *JAMA Network Open*.

eTable 10. Cox Regression Analyses for Primary Syphilis

Outcome	Hazard Ratio	95% Confidence Interval	P Value
Death	3.49	0.86–14.17	0.08
Aortic Aneurysm/Dissection	2.13	0.53–8.62	0.28
Hemorrhagic Stroke	NA	—	0.99
Aortic Regurgitation	NA	—	0.99
Ischemic Stroke	0.52	0.13–2.08	0.35
Venous Thromboembolism	11.31	3.50–36.49	<0.001
Acute Myocardial Infarction	0.36	0.05–2.56	0.31
Peripheral Artery Disease	0.76	0.19–3.05	0.70
Heart Failure	0.34	0.09–1.37	0.13
Atrial Fibrillation	0.32	0.04–2.25	0.25

Table 10: Multivariable Cox regression analyses evaluating associations between primary syphilis and cardiovascular outcomes

© 2026 Tsakiris E et al. *JAMA Network Open*.

eTable 11. Cox Regression Analyses for Syphilis, Unspecified

Outcome	Hazard Ratio	95% Confidence Interval	P Value
Death	2.97	1.92–4.58	<0.001
Aortic Aneurysm/Dissection	1.32	0.86–2.02	0.19
Hemorrhagic Stroke	1.78	1.09–2.90	0.02
Aortic Regurgitation	1.53	0.79–2.97	0.21
Ischemic Stroke	1.25	1.01–1.56	0.04
Venous Thromboembolism	1.26	0.59–2.72	0.55
Acute Myocardial Infarction	1.04	0.80–1.37	0.75
Peripheral Artery Disease	1.08	0.82–1.43	0.58
Heart Failure	0.86	0.70–1.05	0.13
Atrial Fibrillation	0.67	0.50–0.91	0.01

Table 11: Multivariable Cox regression analyses evaluating associations between unspecified syphilis and cardiovascular outcomes

© 2026 Tsakiris E et al. *JAMA Network Open*.

eTable 12. Cox Regression Analyses for Syphilis, Early Latent

Outcome	Hazard Ratio	95% Confidence Interval	P Value
Death	NA	—	0.99
Aortic Aneurysm/Dissection	NA	—	0.99
Hemorrhagic Stroke	NA	—	0.99
Aortic Regurgitation	NA	—	0.99
Ischemic Stroke	0.56	0.08–3.97	0.56
Venous Thromboembolism	NA	—	0.99
Acute Myocardial Infarction	2.29	0.74–7.14	0.15
Peripheral Artery Disease	0.81	0.11–5.80	0.83
Heart Failure	0.36	0.05–2.54	0.30
Atrial Fibrillation	1.40	0.35–5.59	0.63

Supplemental Table 12. Multivariable Cox regression analyses evaluating associations between early latent syphilis and cardiovascular outcomes.

© 2026 Tsakiris E et al. *JAMA Network Open*.

eTable 13. Cox Regression Analyses for Syphilis, Late Latent

Outcome	Hazard Ratio	95% Confidence Interval	P Value
Death	4.08	2.37–7.00	<0.001
Aortic Aneurysm/Dissection	2.41	1.41–4.11	0.001
Hemorrhagic Stroke	1.94	0.94–4.01	0.07
Aortic Regurgitation	1.65	0.59–4.56	0.38
Ischemic Stroke	1.82	1.34–2.47	<0.001
Venous Thromboembolism	0.93	0.23–3.84	0.91
Acute Myocardial Infarction	1.36	0.91–2.02	0.12
Peripheral Artery Disease	1.37	0.90–2.07	0.13
Heart Failure	1.12	0.83–1.51	0.47
Atrial Fibrillation	0.79	0.48–1.28	0.33

Supplemental Table 13. Multivariable Cox regression analyses evaluating associations between late latent syphilis and cardiovascular outcomes.

© 2026 Tsakiris E et al. *JAMA Network Open*.

eFigure 1. Kaplan Meier Survival Curve for Heart Failure, Primary Analysis

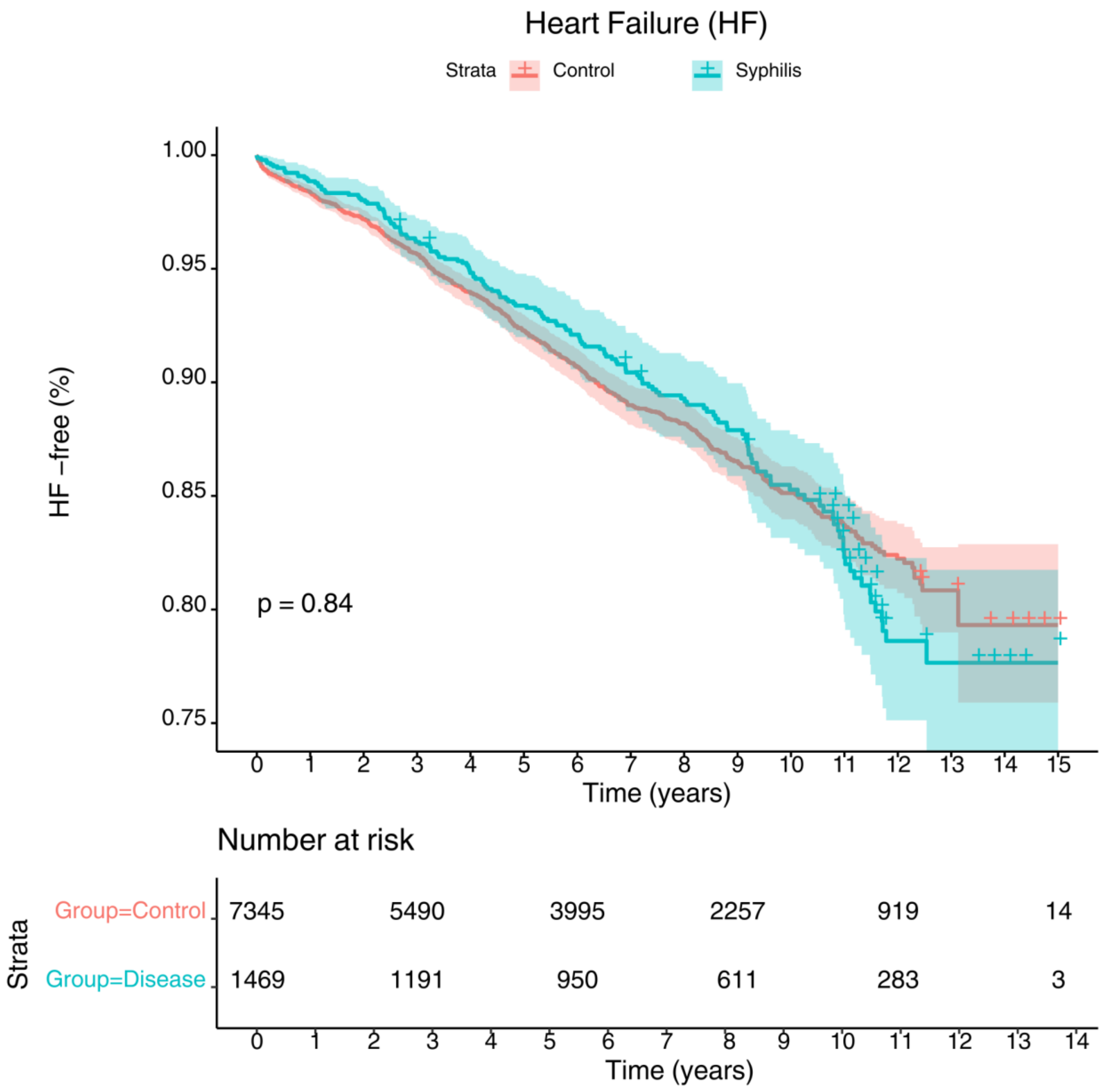

Supplemental Figure 1. Kaplan–Meier curves for heart failure (HF) in patients with syphilis compared with matched controls. The cumulative incidence of HF was similar between groups, with no significant difference in HF-free survival over follow-up (p = 0.84).

© 2026 Tsakiris E et al. *JAMA Network Open*.

eFigure 2. Kaplan Meier Survival Curve for Atrial Fibrillation, Primary Analysis

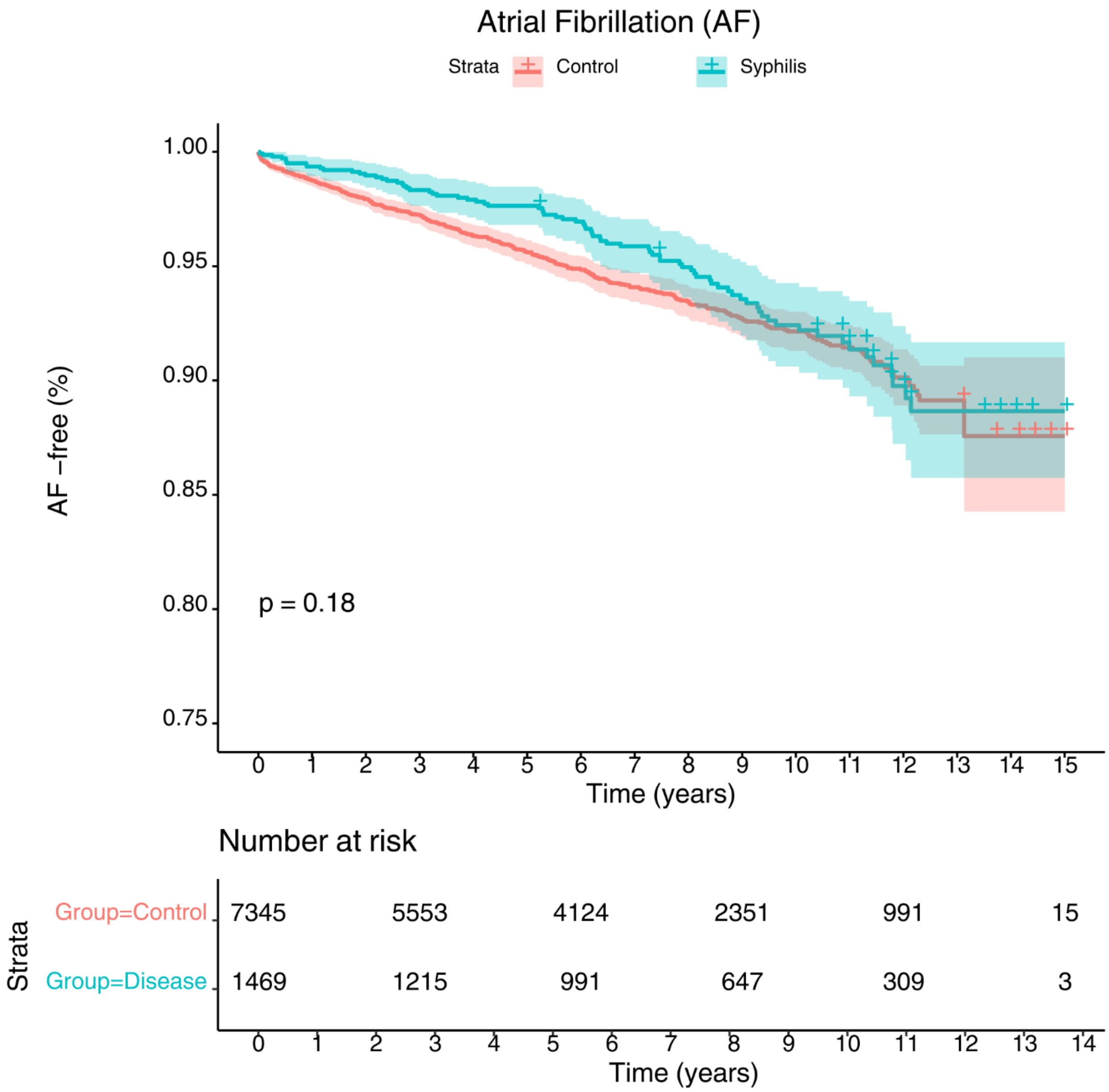

Supplemental Figure 2: Kaplan–Meier curves for atrial fibrillation (AF) in patients with syphilis compared with matched controls. Although the syphilis group appeared to have a higher incidence of AF, the difference in AF-free survival between groups did not reach statistical significance (p = 0.18).

© 2026 Tsakiris E et al. *JAMA Network Open*.

eFigure 3. Kaplan Meier Survival Curve for Aortic Regurgitation, Primary Analysis

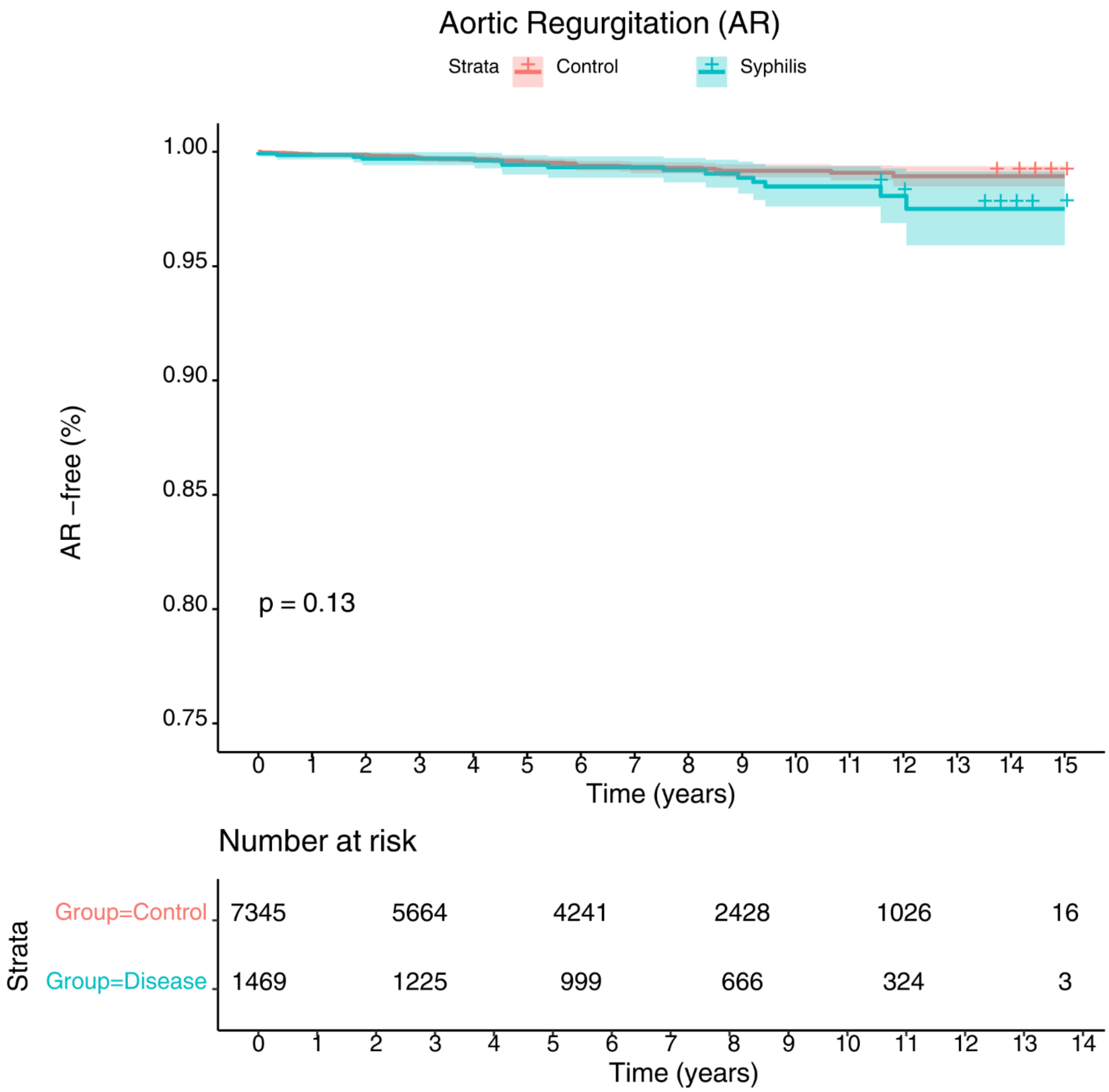

Supplemental Figure 3: Kaplan–Meier curves for aortic regurgitation (AR) in patients with syphilis compared with matched controls. There was no statistically significant difference in AR-free survival between patients with syphilis and controls (p = 0.13).

© 2026 Tsakiris E et al. *JAMA Network Open*.

eFigure 4. Kaplan Meier Survival Curve for Hemorrhagic Stroke, Primary Analysis

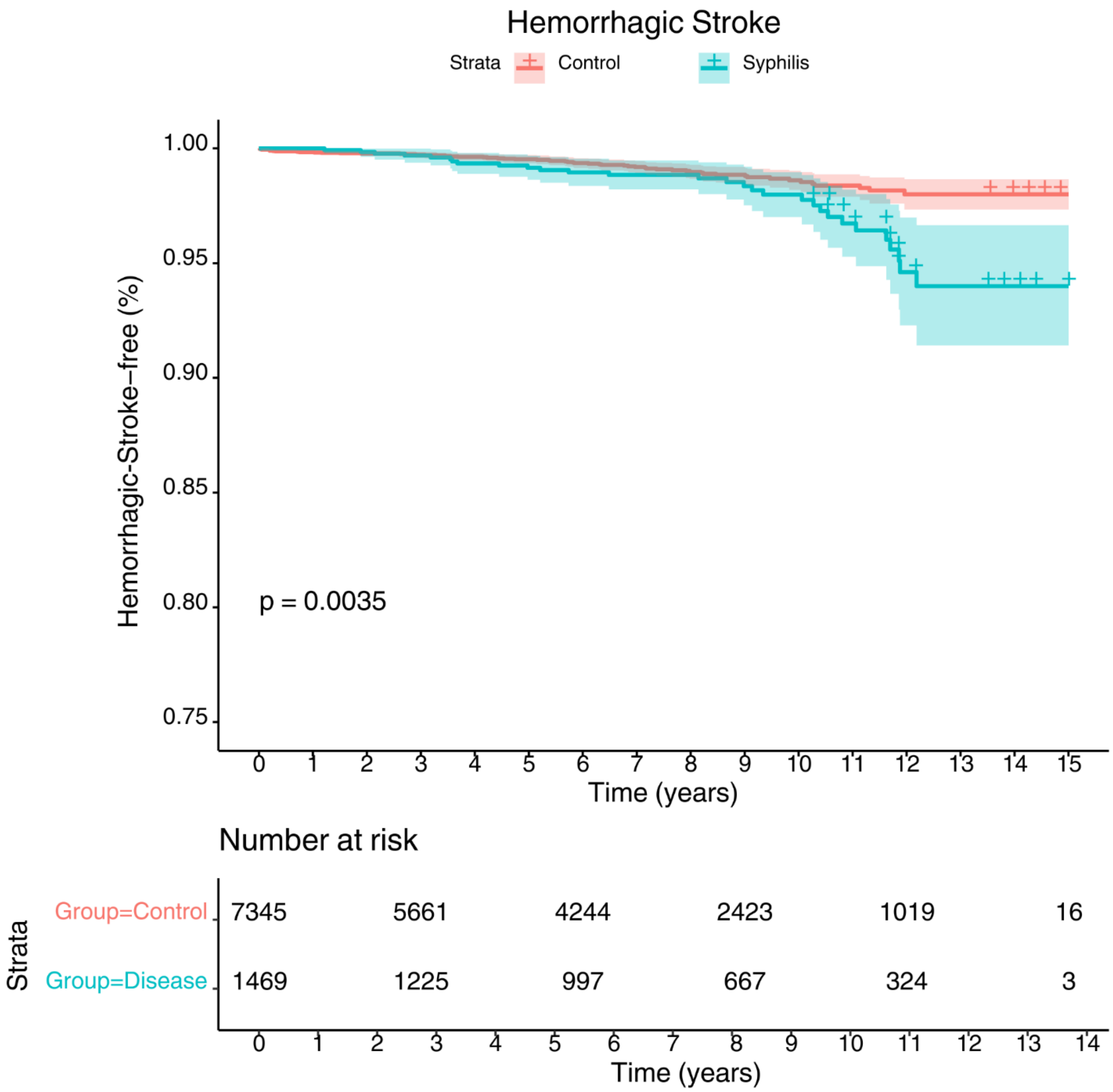

Supplemental Figure 4. Kaplan–Meier curves for hemorrhagic stroke in patients with syphilis compared with matched controls. Patients with syphilis demonstrated a significantly higher risk of hemorrhagic stroke, with lower stroke-free survival compared with controls (p = 0.0035).

© 2026 Tsakiris E et al. *JAMA Network Open*.

eFigure 5. Kaplan Meier Survival Curve for Venous Thromboembolism, Primary Analysis

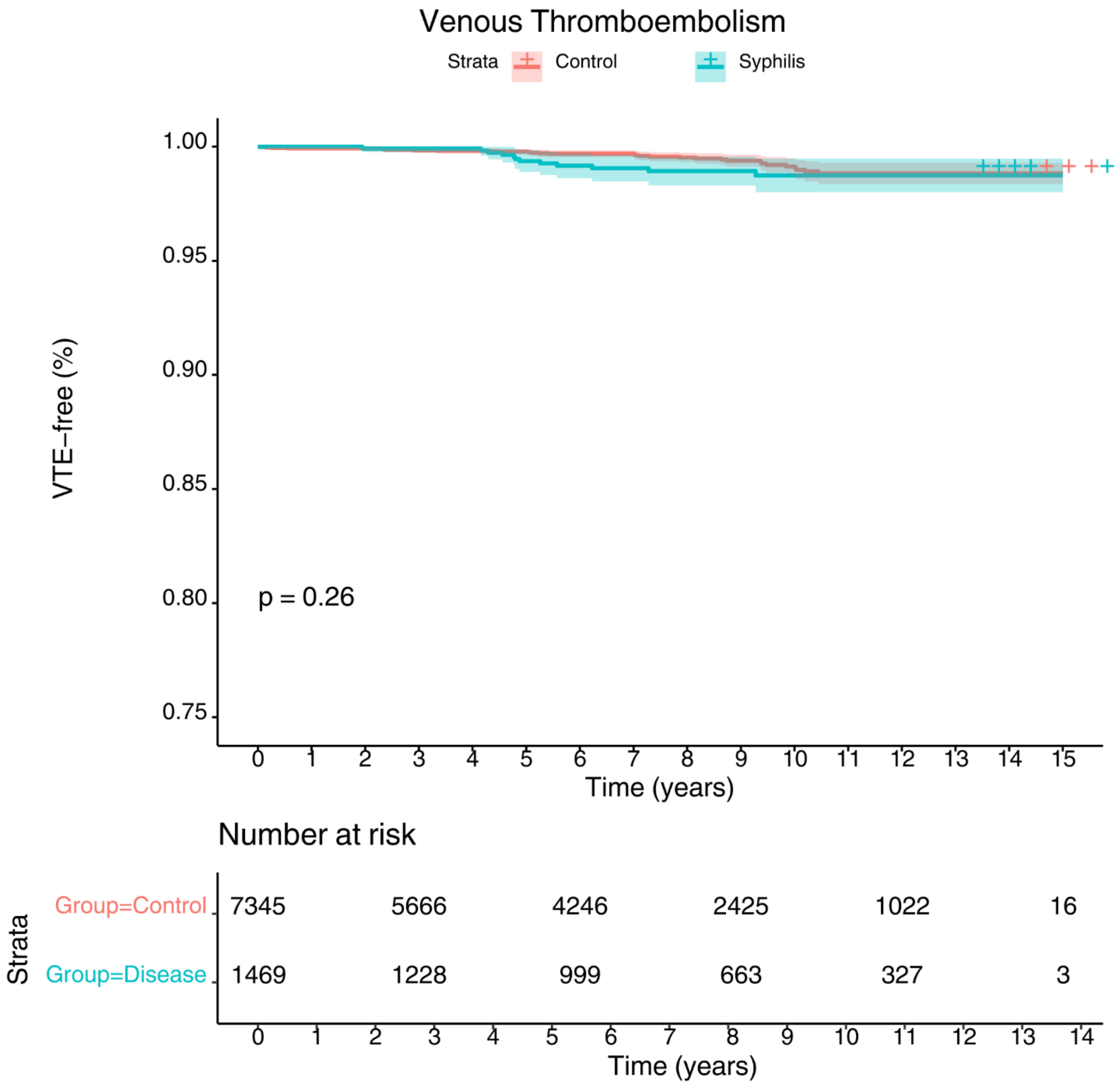

Supplemental Figure 5: Kaplan–Meier curves for venous thromboembolism (VTE) in patients with syphilis compared with matched controls. There was no statistically significant difference in VTE-free survival between patients with syphilis and controls (p = 0.26).

© 2026 Tsakiris E et al. *JAMA Network Open*.

eFigure 6. Kaplan Meier Survival Curve for Mortality, Primary Analysis

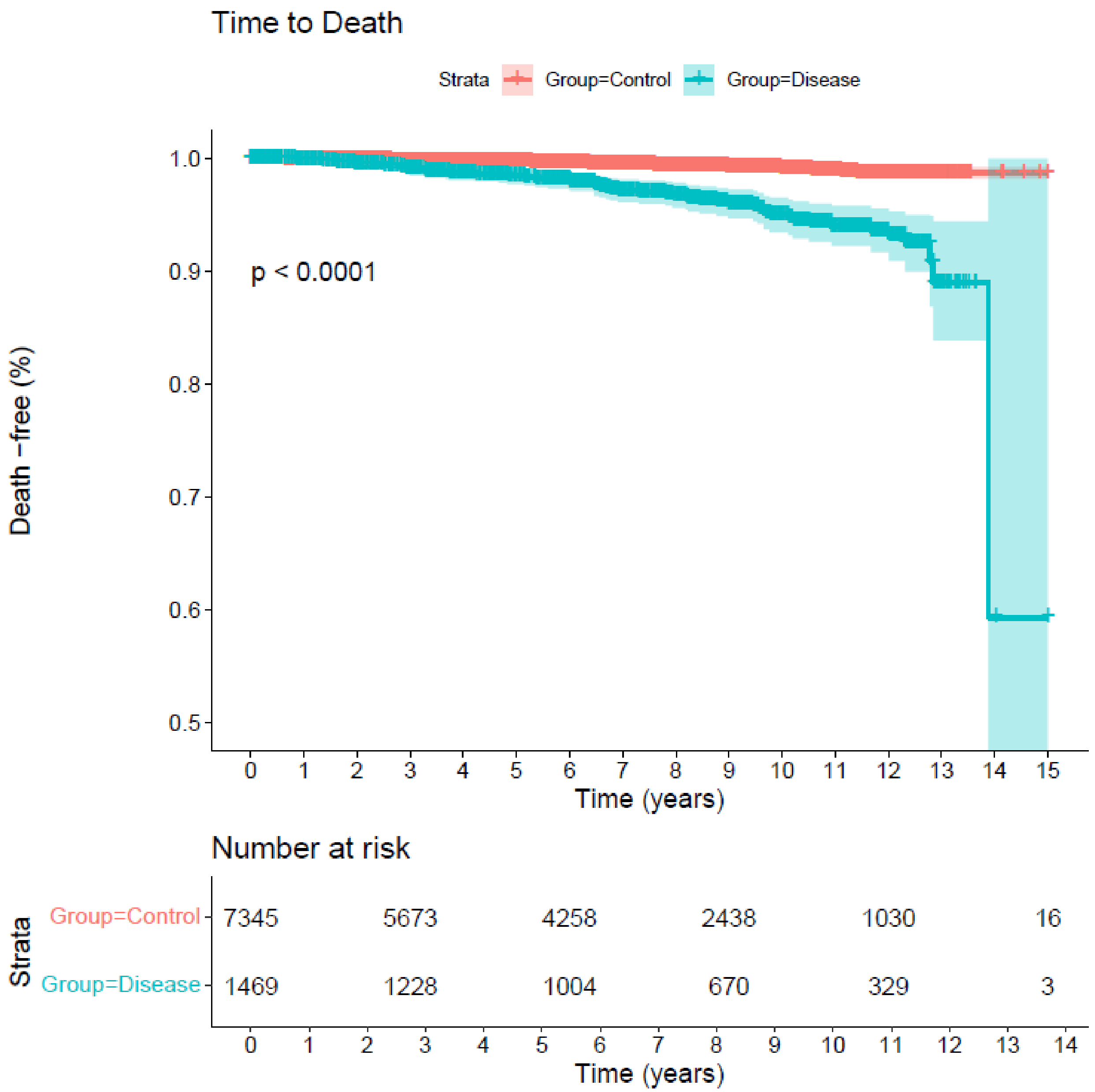

Supplemental Figure 6: Kaplan–Meier curves for mortality comparing patients with syphilis vs controls. Patients with syphilis experienced a statistically significant higher rate of mortality (p<0.001)

© 2026 Tsakiris E et al. *JAMA Network Open*.

eFigure 7. Forest Plot of Estimated Hazard Ratios, HIV Subanalysis

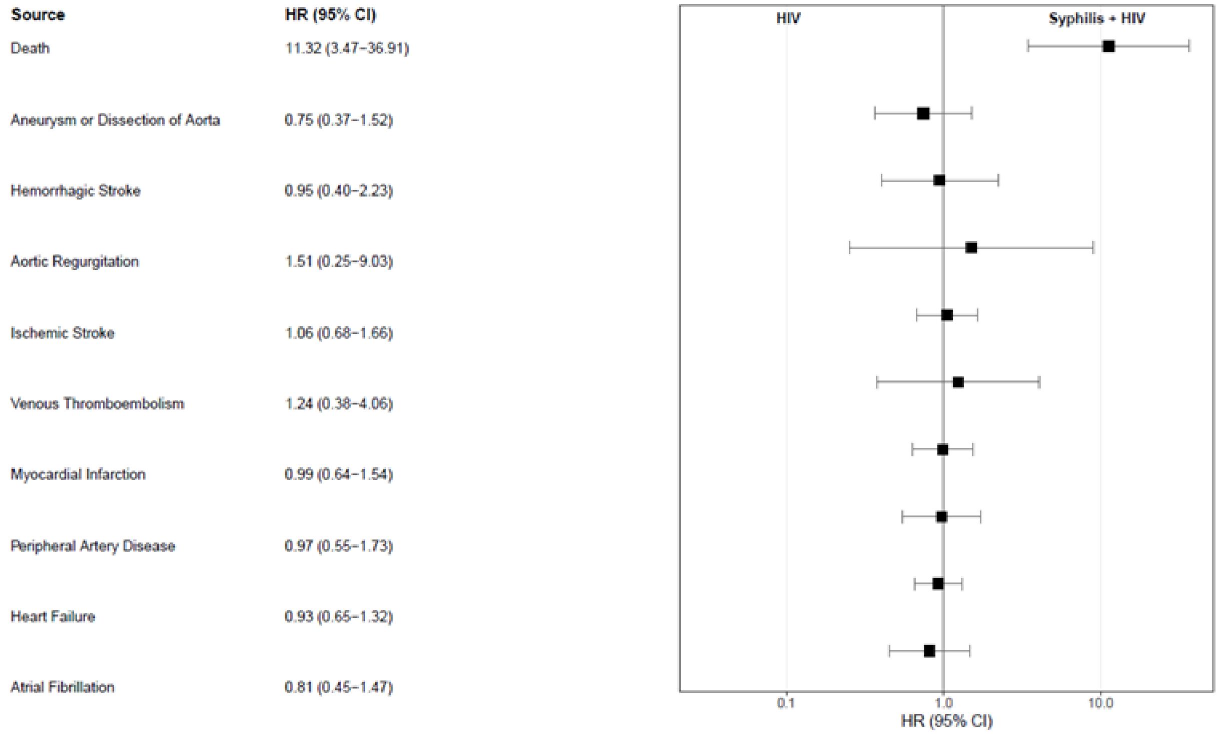

Supplemental Figure 7: A forest plot displaying estimated hazard ratios, 95% confidence intervals and p values of association of syphilis infection with primary outcomes in the subgroup analysis of patients comorbidly infected with Human Immunodeficiency Virus.

© 2026 Tsakiris E et al. *JAMA Network Open*.
