# Supplementary material for: Adverse Cardiovascular Outcomes in Patients With Syphilis
Source: JAMA Netw Open. 2026 Apr 13;9(4):e266771. doi: 10.1001/jamanetworkopen.2026.6771 (PMC13077513; doi:10.1001/jamanetworkopen.2026.6771)
Supplement: Supplement 2. — Data Sharing Statement [file jamanetwopen-e266771-s002.pdf]

Data Sharing Statement

Tsakiris. Adverse Cardiovascular Outcomes in Patients With Syphilis. *JAMA Netw Open*. Published April 13, 2026. doi:10.1001/jamanetworkopen.2026.6771

Data
Data available: No

Additional Information
Explanation for why data not available: De-identified data can be shared upon reasonable request to the corresponding author.
